# Impact of Informal Care on Health Care Utilization Among Older People in China

**DOI:** 10.1093/geroni/igab046.1289

**Published:** 2021-12-17

**Authors:** Yixiao Wang

**Affiliations:** King's College London, London, England, United Kingdom

## Abstract

Population aging has become a challenge to long-term care and health care for the society. Using China as a case study, this paper assesses allocative efficiency of resources in informal care and health care, to explore the effectiveness of the policy, i.e., encouraging informal care as a more cost-effective way to reduce public health care spending. Drawing data from the 2011, 2014, and 2018 waves of the Chinese Longitudinal Healthy Longevity Survey, this study examines the impact of informal care on utilization of health care as well as amount of health care expenditures among older people with functional limitations in China. Using random effects model with instrumental variable approach, our findings suggest that informal care significantly reduces the utilization of health care, primarily by reducing the utilization of outpatient care. However, informal care significantly increases the amount of inpatient care expenditures for inpatient care users. We do not observe significant association between informal care and amount of outpatient care expenditures for outpatient care users. This study highlights a pressing need for the Chinese government to support informal caregivers by taking economic values of informal caregiving into consideration, and to improve efficiency in inpatient care by a more integrated resource allocation mechanism

